# Evolution, the Immune System, and the Health Consequences of Socioeconomic Inequality

**DOI:** 10.1128/msystems.01438-21

**Published:** 2022-03-14

**Authors:** Graham A. W. Rook

**Affiliations:** a Centre for Clinical Microbiology, Department of Infection, UCL (University College London), London, United Kingdom; University of Maine

**Keywords:** brain development, evolutionary biology, immune dysfunction, immune regulation, microbiota, socioeconomic status

## Abstract

Healthy development and function of essentially all physiological systems and organs, including the brain, require exposure to the microbiota of our mothers and of the natural environment, especially in early life. We also know that some infections, if we survive them, modulate the immune system in relevant ways. If we study the evolution of the immune and metabolic systems, we can understand how these requirements developed and the nature of the organisms that we need to encounter. We can then begin to identify the mechanisms of the beneficial effects of these exposures. Against this evolutionary background, we can analyze the ways in which the modern urban lifestyle, particularly for individuals experiencing low socioeconomic status (SES), results in deficient or distorted microbial exposures and microbiomes. Thus, an evolutionary approach facilitates the identification of practical solutions to the growing scandal of health disparities linked to inequality.

## PERSPECTIVE

It is well known that life expectancy and many health problems discussed below are closely linked to socioeconomic factors (1–3). Interestingly, many aspects of life for individuals experiencing low socioeconomic status (SES) lead to diminished exposures to microorganisms that play important roles in the development and function of numerous organs, including the crucial establishment of the regulatory pathways of the immune system, and the establishment of a biodiverse symbiotic microbiota. In addition to SES-linked differences in microbial exposures, other SES-linked factors, such as pollution, diet, exposure to stress, smoking, and vaccine hesitancy, cause changes to the eventual composition of the microbiome. Thus, at least some of the health deficit in low-SES populations is likely to be mediated via changes in microbial exposures and microbiomes (4). There is some direct epidemiological evidence for links between SES and the gut microbiome. Fecal samples were collected from American subjects during sigmoidoscopy, while the subjects’ addresses were used to generate a composite indicator of SES, based on income, education, employment, and home value. A positive correlation was found between SES and the alpha-diversity of the colonic microbiome (5). Recent studies have concentrated on children. A study of the gut microbiomes of children with a mean age of 4.5 years found that the bacterial composition was significantly influenced by the SES of parents as determined by educational level (6). Similarly, a study of fecal samples from a subset (*n* = 1,672) of a large cohort of British twins for whom SES data were available (7), and another study of 139 Arab children of various SESs in Israel (8), found significant relationships between SES and fecal 16S rRNA microbiome composition. Examination of the data from these studies in terms of bacterial taxonomy is unhelpful, perhaps because dietary factors such as meat eating correlate differently with SES in different communities. Nevertheless, the data indicate cumulatively that SES modulates the microbiota in adults and importantly in children. This paper uses an evolutionary approach to outline some of the ways in which low SES interrupts and/or distorts microbial exposures.

## EVOLUTION OF RELATIONSHIP TO MICROBES

Eukaryotic life evolved about 1.5 billion years ago when an organism resembling an alphaproteobacterium started to live inside another organism (9). This suggests that humans, like all eukaryotic life forms, evolved from a blend of 2 or more microbes. Moreover, about 65% of human genes appear to have originated in bacteria, archaea, and eukaryotic microbes (10), including, for example, the genes enabling synthesis of neurotransmitters (11) and the proteins that mediate pyroptosis (12).

This early microbial origin of the ancestral forms of many genes is a fundamentally crucial point in another context that is important for this review. If early versions of so many genes evolved long ago in microbes, then essentially all life forms are constructed from closely related building blocks. This may explain why exposure to a very broad diversity of microorganisms, even if entirely harmless, can prime memory T cells that recognize previously unencountered pathogens such as HIV (13) or the COVID-19 virus (14).

### Inevitable exposures to microorganisms.

So, we evolved from microorganisms, but we also evolved with them. Expressed in terms of the carbon they contain, bacteria are second only to plants in terms of total biomass on our planet, and the biomass of bacteria and archaea is about 1,200× greater than the total biomass of all humans (15). So, massive exposure to microorganisms throughout evolution was inevitable, and evolution tends to convert the inevitable into a necessity. Thus, a complex symbiotic microbiota is a necessity for vertebrates, though it is possible that some invertebrates with short life spans have not evolved this requirement (16).

The complex microbiota of vertebrates is necessary, both in order to populate the symbiotic microbiotas, notably in the gut, and in order to provide signals and data described below (17–20). The resident microbiota also provides metabolites with profound effects on our physiology and on the development of most, perhaps all, organs (21). It has been estimated that 30% or more of the small molecules in our peripheral blood are products of microbial metabolism (22).

Early in evolution, organisms that entered the gut were separated from the host by a chitin barrier (23). This structure persists in annelids and arthropods, but in mammals the chitin layer is lost and several mucus layers nourish a much more complex assembly of microorganisms, many of which adhere to the mucus and modulate the function of the underlying cells (23). This parallels the situation in plants where organisms are attracted by molecules secreted from the roots. These molecules nourish the organisms which take part in symbiotic two-way signaling and exchange of nutrients (24). Moreover, in addition to the well-known physiological roles of microbial products such as short-chain fatty acids (SCFA), bile salt metabolites, and metabolites of tryptophan and tyrosine, there is now evidence that small noncoding RNAs present in exosomes (membrane-bound extracellular vesicles [EV]) derived from the host gut epithelium and similar small noncoding RNAs present in membrane vesicles (MVs) from Gram-positive bacteria or outer membrane vesicles (OMVs) from Gram-negative bacteria may be involved in 2-way mutual gene regulation (25, 26; reviewed in reference 27).

## EVOLUTION OF THE IMMUNE SYSTEM

**FIG 1:**
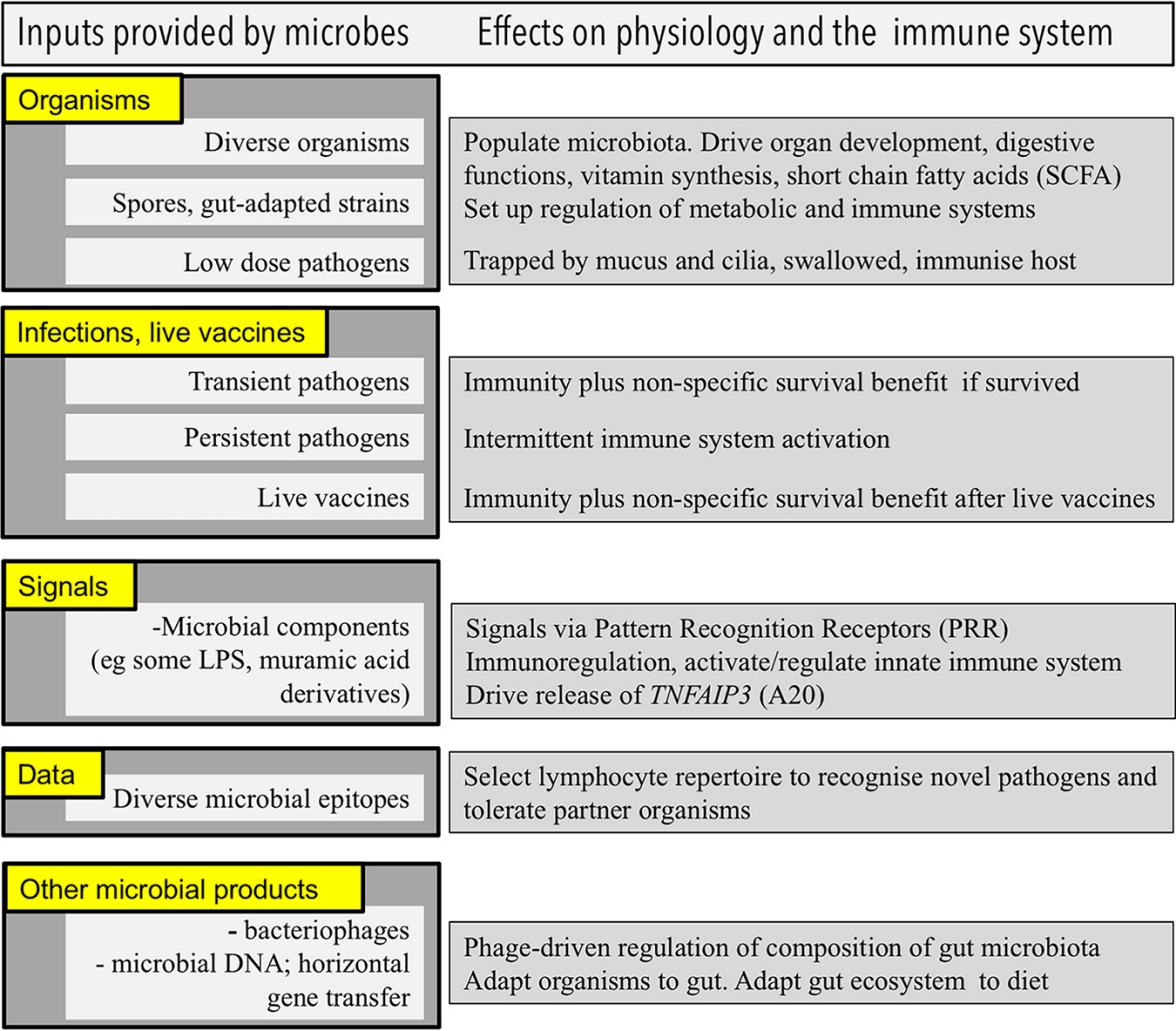
A simple classification of the variety of inputs provided by microbes that influence organ development, physiology, metabolism, and the immune system. References are in the main text. LPS, lipopolysaccharide.

This evolutionary background helps us to understand the evolution of the immune system. The role of symbiosis in the evolution of the immune system has been extensively covered elsewhere (28–31) and is reviewed very briefly here. The development of the complex vertebrate microbiota necessitated an upgrading of the immune system that would enable it to “manage” the very large and diverse community of organisms in the gut while simultaneously protecting the host from pathogens. The existing innate immune system recognized microorganisms by using germ line-encoded pattern recognition receptors (PRR). The repertoire of these receptors is small and cannot be expanded rapidly enough to cope with fast-evolving microbes. Moreover, a large expansion of PRR genes would constitute a problematical increase in genetic complexity (32). The adaptive immune system evolved to solve these problems. It creates a large number of lymphocytes by somatic mutation of the receptors of B cells and T cells, so that each new individual generates a repertoire of novel lymphocyte clones. Some of these lymphocyte clones will be autoreactive while others will recognize nothing at all, and so the clones that express them must be eliminated. Then, microbial exposures are absolutely necessary for further education and selection of the clones to be retained (33, 34). For example, some clones will recognize organisms that need to be tolerated (such as most of the gut microbiota derived from the mother) and exposure to the gut microbiota can cause them to become regulatory cells. Microbial metabolites and components help to expand these cell populations (Fig. 1) (35, 36). Similarly, exposure to a very diverse range of microorganisms from the environment can prime memory T cells that by chance recognize even novel organisms that the individual has never previously encountered, such as HIV or the pathogen causing COVID-19 (13, 14). Some of the types of organisms and information that microbial exposures supply to the immune system are listed in Fig. 1 and discussed further in sections below.

## EVOLUTION AND NECESSARY MICROBIAL EXPOSURES

**FIG 2:**
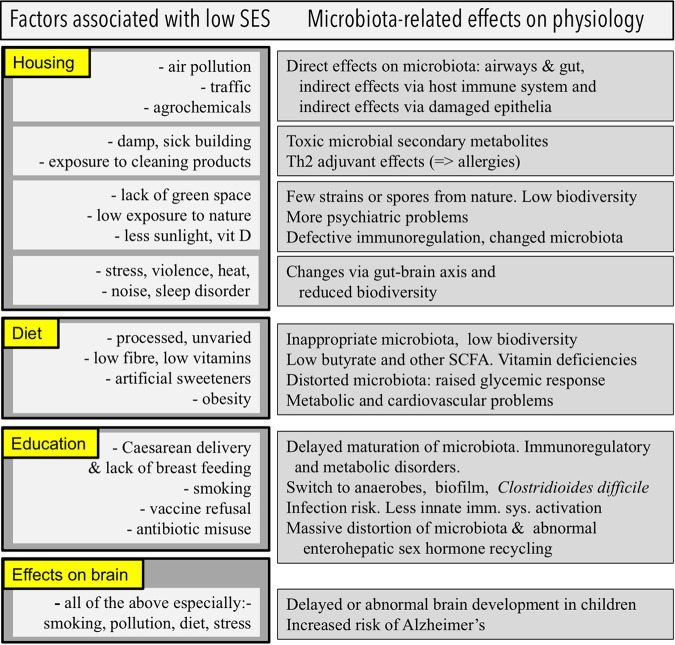
Factors associated with low SES that have profound effects on microbial exposures and on the composition of the microbiota and the consequent microbiota-mediated effects on health. References are in the main text.

Humans evolved in small hunter-gatherer groups close to streams, rivers, lakes, or the sea. Exposure to the microbiota of family, animals, and the environment was therefore inevitable, while early humans were not exposed to anything like the microbiota of the modern home. Similarly, exposure to infections was mostly limited to organisms that could persist in small groups and would have excluded the “crowd infections” discussed below (37). SES-associated factors that limit necessary microbial exposures are summarized in Fig. 2 and discussed below.

### Mother/family.

Mother and siblings are major sources of the microorganisms that establish the infant’s microbiome and provide signals driving development of the infant’s immune and metabolic systems (38). The major lifestyle factors that reduce this transfer and correlate with increased immunoregulatory disorders are caesarean deliveries, lack of breastfeeding, and lack of mother-baby intimacy (38–40), together with antibiotic use and poor diet, which are discussed in detail below. A recent very large epidemiological study revealed that caesarean section was associated with an increased risk of asthma, laryngitis, gastroenteritis, ulcerative colitis, celiac disease, lower respiratory tract infection, and juvenile idiopathic arthritis (41), all of which are linked to disturbed mucosal immunity.

Lack of breastfeeding and caesarean deliveries can be associated with low SES in high-income contexts, but there is likely to be variation in different cultures. A random sample of 10,519 women delivering live births in California between 1999 and 2001 revealed that lack of breastfeeding was strongly associated with low SES and particularly with low educational level (42). However, low SES may correlate with increased breastfeeding in developing countries in South America or Africa (43, 44).

Similarly, caesarean deliveries are not available everywhere, but in France, a high-income country, where they are readily available, women of low SES are more likely to have caesarean deliveries and less likely to participate in prenatal education, so perhaps they are less aware of the disadvantages (45).

Maternal diet, obesity, gestational weight gain, and gestational diabetes mellitus are additional factors that modulate the nature of the microbiota that is transferred to the fetus and neonate. This topic has been extensively reviewed elsewhere (46, 47), and overweight and other aspects of diet are discussed below.

### Microbiota of the home.

Much of the literature on the badly named “hygiene hypothesis” focuses on the microbiota of the home, but an evolutionary approach tells us that the microbiota of the modern home, constructed with synthetic and biocide-treated materials, does not resemble the microbiota to which our ancestors were exposed (48, 49). Caves, shelters, and premodern houses built with untreated natural materials such as timber and thatch and rendered with straw, mud, dung, and clay would have housed a microbiota similar to that of the natural environment, even when damp and deteriorating. In sharp contrast, modern houses, especially if remote from the natural environment, develop an unusual microbiota (50). This microbiota can become toxic if the modern home is damp and deteriorating, as homes of low-SES residents frequently are, because it can include bacteria and fungi that produce secondary metabolites toxic to humans, resulting in “sick building syndrome” (51–54) and a greater risk that children will be hospitalized for respiratory infections (55). From an evolutionary point of view, it is therefore unlikely that the unnatural microbiota of the modern home is a necessary exposure for infants, except to the extent that it also contains some microbiota from mother, family, and nature.

### Th2 adjuvanticity of cleaning agents.

Why then do some studies find epidemiological links between cleaning the home and allergic disorders (56), whereas other extremely detailed studies do not (57)? The probable explanation for this conflict has emerged recently and again points to the major role of SES. It is well established that cleaning personnel exposed every working day to agents such as detergents and quaternary ammonium compounds, often used as sprays, are at risk of developing asthma (58). Infants from low-SES homes are much more likely to be exposed to aerosols of these cleaning materials than infants from large, wealthy homes where cleaning is undertaken by paid employees rather than by a mother who is simultaneously caring for small children. It is now understood that intake of toxic materials via the airways or gut can cause local cell death (59) and increased epithelial permeability (60). Such “danger signals” activate a Th2 response to whatever antigens happen to be present at the time, and these will often be common aeroallergens or foods (59, 60). Then, the next time this allergen is taken in, an allergic response occurs, because the allergen is being treated as a proxy for the toxic material. Laboratory studies of adjuvants that drive Th2 responses have demonstrated the probable mechanism of this Th2 adjuvant effect of domestic cleaning agents (61, 62), and this is discussed and illustrated elsewhere (63).

### Microbiota of the natural environment.

On the other hand, as an evolutionary approach would suggest, the microbiota of the home does become beneficial when it resembles that of the natural environment, at least where asthma and other disorders associated with faulty immunoregulation are concerned (64–67). Much of the best evidence has come from studies of farming environments (17), but a recent well-controlled intervention experiment has demonstrated that deliberately exposing children to biodiversity from the natural environment in their school playgrounds results in changed microbiome and increased peripheral blood biomarkers of immunoregulation (68).

The direct evidence for the protective effect of exposure to the natural environment is weaker for the other chronic inflammatory disorders, but there is suggestive evidence for inflammatory bowel diseases (69) and for autoimmune diseases (70). It is worth noting that autoantibodies, including those that neutralize type I interferons (IFNs), are associated with breakthrough COVID-19 infections, mortality, and distinct patterns of postacute COVID-19 syndrome (PACS; also known as “long COVID”) (71). PACS is accompanied by unusual composition of the microbiota (72). Moreover, in some contexts COVID-19 mortality is associated with low SES (73). So, perhaps SES-related defects in the microbiota are linked to susceptibility to COVID-19.

There is also strong evidence that exposure to the natural environment protects from metabolic syndrome, obesity, and cardiovascular disease, which are health problems that plague modern humans, particularly those of low SES (74, 75).

Psychiatric disorders have also increased in rich urban societies. A study of approximately 1 million Danish citizens found that the risk of a wide variety of mental illnesses in later life was 55% higher in those most deprived of green space in childhood, compared to those who had lived close to green space during childhood (76), and similar results for depression and anxiety were reported in a large study in the Netherlands (77). It is not certain that a similar link between lack of exposure to green space in childhood and subsequent psychiatric disease would be found in developing countries. Some of the effect seen in the Danish study was associated with low SES (76). Clearly, there are many possible explanations for the relationship seen in wealthy countries, but evidence suggesting roles for the microbiota and SES in the development, function, and pathology of the brain is presented below.

### (i) Spores from the natural environment.

Spores of gut-adapted strains are an important input from the environment. About 60% of the bacterial genera in the gut make spores, but many of these are strictly anaerobic (78, 79). Therefore, direct transmission from mother via the air is limited, and transmission as spores via feces and the natural environment facilitates necessary transfer from one individual to another (78, 79). Spore-forming organisms may be among the components of the child’s microbiota that accumulate slowly throughout infancy and are still accumulating at 5 years of age (80). They are important because they drive formation of SCFA (81) and proliferation of regulatory T cells (Treg) (35, 70, 82).

### (ii) Bacteriophages from the natural environment.

There are about 10^9^ phages/g of soil (83) and in drinking water, so vast numbers of bacteriophages are taken in every day and form ∼90% of the gut virome (84). These phages can modulate the metabolism, antibiotic resistance, and turnover of gut bacteria and archaea (85), so they must influence the composition of the microbiota. Moreover, it is thought that every day about 30 billion phages from the gut enter human tissues and circulation (85), and since they can evoke antibody responses, there may be complex interactions between the host immune system, phages, and gut microbial turnover. It is interesting that Clostridioides difficile infection can be treated using 0.2-μm-filtered, bacterium-free fecal material (86), raising the possibility that phages were involved in the treatment.

### (iii) Horizontal gene transfer from environmental microbial DNA.

A large proportion of the genes found in the human microbiome seem to have been acquired from organisms in the environment via horizontal gene transfer (87–90). This emphasizes another crucial role of environmental microbiota as a source of genetic diversity, allowing the human microbiota to adapt to a changing diet (88, 91) or allowing environmental strains to adapt to the human gut (92).

### Infections and immunoregulation.

Up to this point, this paper has considered exposure of mostly harmless microorganisms from mother, home, and environment. But what about infections?

### (i) Are the “crowd infections” immunoregulatory?

The original “hygiene hypothesis” suggested that protection from hay fever by contact with older siblings might be due to exposure to the common infections of childhood (93). However, epidemiological studies rapidly demonstrated that these infections do not provide such protection and more often exacerbate allergic conditions of the respiratory system (94, 95) and often actually trigger them (96). Thus, the common infections of childhood do not seem to prime the essential immunoregulatory negative feedback mechanisms that are failing in the chronic inflammatory disorders such as allergies and autoimmunity. Moreover, these infections are mostly “crowd infections” that did not exist in human populations until large human communities evolved so it is unlikely that humans are in a state of evolved dependence on them. (Measles, for example, might not have emerged until the 11th or 12th century (97), or perhaps a few hundred years earlier (98)). Crowd infections are therefore more relevant to nonspecific innate immune system activation that to immunoregulation, a role now at least partly replaced by the nonspecific effects of vaccines discussed below.

### (ii) Immunoregulatory “old infections.”

In contrast to the “crowd infections,” several “old infections” that establish lifelong carrier states or subclinical infections were able to survive within small hunter-gatherer groups and were present in human populations before the migrations out of Africa (37) and coevolved and spread over the globe with human populations (37, 99, 100).

*(a)* Helicobacter pylori. Helicobacter pylori is a persistent “old infection” that might suppress childhood asthma (101). It was associated with low SES in the United Kingdom in the 1990s (102), and it is still associated with low SES in Cameroon (103). A recent study reported that in humans, in addition to its local effects in the stomach, H. pylori infection caused increased relative abundance of a variety of bacterial species and a notable increase in Candida glabrata and several unclassified fungi. The authors suggest that these changes might influence mucosal barrier integrity in the lower intestine and contribute to the risk of colorectal carcinoma (104). In mouse models H. pylori causes expansion of Treg subsets expressing CXCR3 or RORγt and demethylation of the FOXP3 locus (105). H. pylori has been carried by humans throughout much of our evolutionary past, but antibiotic use has caused H. pylori seroprevalence to fall below 10% in native-born citizens of Western urbanized countries (101). In modern societies, any possible immunoregulatory benefit of Treg induction by H. pylori in the context of asthma is now overshadowed by the multiple pathologies associated with it.

*(b) Helminths.* Helminth infections, because they persist for a lifetime, tend to downregulate the immune system in order to avoid immunopathology that would endanger the host. Some authors believe that lack of helminths is contributing to the rise in immunoregulatory disorders and that we need to reintroduce helminth infections into contemporary citizens (106). However, the multitude of helminth species, the diverse tissue sites involved, the diverse range of immunoregulatory mechanisms employed (107), and the greatly varying prevalence of infection (108) make it unlikely that we are in a state of evolved dependence on helminths (109). I have stated elsewhere that evolution turns the inevitable into a necessity (31). However, since there was nothing inevitable or constant about helminth infection during human evolution, it is unlikely that there is a germ line-encoded evolved necessity for helminth infection. It is more probable that if, and only if, a mother and her infant are infected, then there is epigenetic modification of the developing child’s immune system tailored to the helminths that are present. Interestingly, clinical trials of the administration of helminths to treat multiple sclerosis (MS) have worked convincingly in Argentina, where the subjects would have been infected with the same endemic helminths in childhood (110). But such trials consistently fail in developed countries where helminths have not been endemic for generations, so the population does not have an epigenetically encoded requirement for helminth-mediated immunoregulation (111, 112). This does not mean that certain individuals suffering from a specific chronic inflammatory disorder for a specific immunoregulatory reason might not benefit from treatment with a helminth that targets that particular mechanism, but without more research on mechanisms, rational selection of the appropriate combination of individual, disorder, and helminth is not possible.

### Infections and immunostimulation.

Some infections are less relevant to anti-inflammatory and immunoregulatory functions discussed above in relation to chronic inflammatory disorders but instead confer nonspecific health benefits by nonspecifically activating the immune system. This has been known since 1936 when Pullinger showed that Mycobacterium tuberculosis conferred resistance to Brucella abortus and suggested that the phenomenon was attributable to activated monocytes (113). Subsequent workers amplified this concept by showing cross-protection between unrelated parasite species, between bacteria and parasites, or between Listeria monocytogenes and influenza virus (114). Sometimes the effect occurs intermittently throughout life when a persistent infection reactivates. Three examples are provided.

### (i) Herpesviruses.

Most adults are latently infected with one or more of the five commonest species (herpes simplex virus 1 [HSV-1], HSV-2, varicella-zoster virus, Epstein-Barr virus, and cytomegalovirus [CMV]). Periodic reactivation can modulate the immune system. For example, mice latently infected with herpesviruses were found to be resistant to infection with Listeria monocytogenes and Yersinia pestis (115). A recent study in the United States found higher rates of seropositivity for HSV-1, HSV-2, and CMV in people of lower SES (116), but it is not clear that the putative immunostimulatory effects overcome the other disadvantages of these infections or of low SES.

### (ii) Tuberculosis.

As observed by Pullinger in 1936 (113), M. tuberculosis exerts nonspecific immunostimulatory effects. Latent tuberculosis infection (LTBI) is often associated with low SES, particularly in developing countries. The latent organisms have been demonstrated by *in situ* PCR in multiple tissues (117). The crucial point is that treating LTBI in individuals who are not HIV infected, despite reducing the incidence of clinical tuberculosis, fails to provide a survival benefit because of increased mortality from other causes (118). This suggests that in this case the putative immunostimulatory effects are significant and beneficial, but interestingly, the benefit can be replicated by Mycobacterium bovis BCG vaccine (see below).

### (iii) Vaccines.

Research since the 1980s, initially in Africa but recently confirmed in high-income countries such as Italy, Denmark, the Netherlands, and the United States, has demonstrated that several live vaccines including measles, polio, and BCG decrease childhood mortality more than can be explained by protection from the targeted infection, apparently by enhancing resistance to unrelated infections (119–121). Similarly, a recent clinical trial demonstrated that vaccinating elderly subjects with BCG can decrease their risk of virus infections (122). This suggests some nonspecific activation of the innate immune system, and it has emerged that these nonspecific vaccine effects operate via NK cells and monocytes (123, 124), as the much earlier work of Pullinger and of Gregorio et al. had suggested (113, 114). This activation of innate immune cells is attributable to epigenetic modification of hematopoietic stem cells (123, 124). Clearly, therefore, vaccine refusal or hesitancy, which is associated with low SES (125, 126), is not only depriving people of protection from the targeted infection but also depriving them of a more general upregulation of the innate immune system.

## SES-LINKED FACTORS THAT FURTHER DISTORT THE MICROBIOTA

In addition to SES-linked variation in microbial exposures outlined above, numerous lifestyle and environmental factors influence the composition of the microbiota, either directly by affecting the microorganisms or indirectly via effects on physiological systems. Some of the most obvious factors are listed in Fig. 2 and described below.

### Pollution and SES.

A recent study of exposure to PM2.5, nitrogen dioxide (NO_2_), and ozone (O_3_) concluded that even at levels officially regarded as safe, exposure to these pollutants for long periods increases mortality (127), and clearly such exposures are greater for citizens of low SES. Moreover, Danish children exposed to high levels of PM2.5 were more likely to develop asthma and persistent wheezing, the risk being increased further if the mother smoked and the parents were of low SES (128). How many of these SES-related effects are mediated by changes to the microbiota?

Air pollutants may act directly on gut epithelial cells to drive intestinal inflammation and changes to the microbiota (129). A recent study of young Californian adults indicated that exposure to air pollution, notably ozone (O_3_), had a large effect on the composition of the gut microbiome, with lower Shannon diversity index and changes in multiple gene pathways (130).

Metabolic dysfunction and type 2 diabetes are increased in populations exposed to air pollution from traffic (131). However, these metabolic disturbances are also associated with abnormal gut microbiomes (132), suggesting that pollution might damage health at least partly via the microbiota.

Pollutants might also act indirectly by changing the microbiota of the natural environment. Polycyclic aromatic hydrocarbons (PAH; derived from coal, crude oil, vehicle exhaust, cigarette and wood smoke, and fumes from asphalt roads) accumulate in urban soils where concentrations can be 10 to 100 times higher than in unpolluted rural soils (133). Another example is the use of unpurified reclaimed wastewater to irrigate parks in China. The levels of antibiotics in this water were sufficient to modify the soil microbiome (134).

In addition to changing the microbial environment, many pollutant chemicals, particularly pesticides to which workers of low SES are more exposed, have direct antibacterial properties and can be detected in the blood or urine of most people (135, 136). For example, glyphosate, which was initially patented as an antimicrobial (137), was detected in 93% of a cohort of pregnant women in the United States (138), though it is unclear whether the levels found would alter the microbiota or exert other harmful effects.

### Smoking and SES.

Smoking, which can be regarded as voluntary exposure to air pollution, is increasingly associated with low education and low SES and with a remarkable range of illnesses including cardiovascular disease, periodontitis, chronic obstructive pulmonary diseases (COPD), Crohn’s disease, and various cancers (139). Low SES is also associated with a lower brain volume and increased risk of dementia. A study using magnetic resonance imaging identified smoking as the major causal factor (140). The fact that smoking is also associated with autoimmune disorders such as MS and rheumatoid arthritis suggests compromised immunoregulation. Are some of these effects mediated via changes in the microbiota? Smoking causes clear changes in the oral, nasopharyngeal, airway, and gut microbiomes (139). Some of these smoking-induced changes in the microbiome could be direct effects on microorganisms of chemicals in smoke, but smoking also modulates both the innate and adaptive immune systems (141), partly via epigenetic modifications (142). I will not review this topic here.

### Diet and SES.

Numerous dietary factors are associated with low SES in modern urban contexts (143–145). Three of the most obvious are outlined here. Artificial sweeteners change the composition of the murine gut microbiome *in vitro*, and this modified microbiota causes glucose intolerance following transfer into germfree mice (146). In a human study, some volunteers developed altered microbiomes after consuming saccharin and also developed an elevated glycemic response. Germfree mice that received transplants of microbiota from these individuals similarly developed altered glycemic responses (146).

Consumption of fructose has increased enormously during the last century, particularly in fruit juices. Excessive consumption may be associated with low SES (147) and is linked to nonalcoholic fatty liver disease, obesity, and diabetes (148). The microbiota of the small intestine metabolizes fructose and so blocks uptake (148), but if too much fructose is consumed, it enters the colon and distorts the microbiome (149). In rat models the resulting metabolic disturbances can be corrected by fecal transplantation or antibiotics (150).

Low vitamin D levels are associated with systemic autoimmune conditions, and vitamin D levels correlate with SES (151). Importantly, vitamin D has effects on the immune system, including promotion of Treg, and modifies the gut microbiome (152, 153), so at least some of the effects of vitamin D deficiency may be mediated via the microbiome.

### Antibiotic misuse and SES.

It is not clear how SES affects misuse and overuse of antibiotics in developing countries because availability and costs are not uniform. However, in countries such as Denmark with good documentation of medical interventions and relatively uniform access to antibiotics, the risk of receiving multiple antibiotic prescriptions in pregnancy and childhood is related to poor parental educational level (154), and in Finland changes in the microbiota have been linked to early-life antibiotic use (155). This is a serious issue since early antibiotic exposure is associated with an increased risk of a large number of disorders, and the risk increases with the number of courses of antibiotics received (156). It is interesting that most or all of these disorders are associated with immunoregulatory problems or with biomarkers of inflammation. The conditions include overweight, obesity (157), childhood-onset asthma and allergic disorders (rhinitis, atopic dermatitis) (158), celiac disease, attention deficit hyperactivity disorder (ADHD), and autism (156).

### Antibiotics, sex hormones, and abnormal development.

A neglected side effect of antibiotic use is disturbance of the enterohepatic circulation of sex hormones. Sex hormones conjugated to sulfate or glucuronide in the liver are secreted into the gut where the microbiota can then deconjugate them and also modify them in functionally significant ways (159). Deconjugated hormones are then reabsorbed, while conjugated forms are mostly lost in the feces, so antibiotics can block reabsorption and modulate circulating levels of sex hormones (159). The interaction of early microbial exposures, sex hormone levels, and progression to autoimmunity has been demonstrated in the NOD mouse model of type 1 diabetes (160). Interestingly, the levels of sex hormone metabolites that are relevant to the risk of breast cancer in postmenopausal women are influenced by the biodiversity of the gut microbiome (161). Early menarche in black and Hispanic girls in the United States seems to be related to low SES, as is early appearance of some secondary sexual characteristics in German children (162). Therefore, modulation of sex steroids by the microbiota of low-SES children might hold the key to the finding that early puberty is associated with increased risk of breast cancer (163).

### Brain development and SES.

In an evolutionary context, the brain and gut work as an intimately connected food-gathering/processing partnership, and there is abundant literature on the role of the microbiome on brain function and development (36, 164–166). Children from low-SES backgrounds tend to show abnormal brain development, with lower cognitive, language, and memory abilities (167), often correlating with differences in development of the areas of the brain associated with these functions, including the surface area of the cortex, the frontal lobe, the temporal lobe, and the hippocampus (168–170).

Some of these SES-related cognitive and structural differences are apparent in the early months of life, and a study using *in vivo* magnetic resonance imaging (MRI)-computed 3-dimensional images of fetal brains found that the volume of the fetal white matter was greater when the mother was of high SES (167, 171, 172). These effects are too early to be attributable to reduced intellectual stimulation in low-SES environments and suggest other environmental factors. Some SES-associated factors already discussed above are known to affect the brain and certainly have major effects on the microbiome. For example, smoking clearly mediates the relationship between low SES and reduced brain volume (140), and antibiotic misuse is associated with autism and ADHD (156). Similarly, a recent meta-analysis reported that most studies (but not all) find that maternal obesity is associated with reduced biodiversity of the infant microbiome (173), and reduced biodiversity has been linked to behavioral abnormalities in a recent prospective cohort study (174).

Evidence that various forms of pollution affect the microbiome was outlined above, and many studies link such pollution to the brain. For example, a study of children born less than 400 m or more than 1,500 m from a polluting major highway revealed that those with higher levels of exposure during the first year of life had significantly reduced cortical thickness and gray matter volume compared to children with low levels of exposure (175), and similar effects have been seen in children exposed to high levels of several air pollutants during pregnancy or childhood (176). There are, however, many other ways in which pollution could affect the developing brain, and more research is needed.

### AD and SES.

As mentioned above, smoking mediates the relationship between low SES and reduced brain volume (140). Similarly, in a cohort of women aged 70 to 89, exposure to PM2.5 was associated with atrophy of gray matter, indicating an increased risk of Alzheimer’s disease (AD) (177). AD is characterized by raised levels of proinflammatory cytokines in the peripheral blood and by activated microglia and innate immune cells in the brain. There is also extraneuronal accumulation of amyloid-β (Aβ), an antimicrobial peptide associated with the innate immune system (178). Could the microbiome be involved in this inflammatory background? It has been reported that the gut microbiome has decreased diversity in patients with Alzheimer’s disease and shows decreased *Firmicutes*, increased *Bacteroidetes*, and decreased *Bifidobacteria* compared to controls without dementia (179). A recent study has suggested microbiome-mediated pathways that might connect this changed microbiome to the inflammation (178).

### Stress and SES.

Many effects of low SES on the composition of the microbiota are difficult to disentangle from the effects of stress, but it is clear that childhood adversity is associated with increased mortality in later life (180), and it is likely that the microbiota is involved. For example, separating rat pups from their mothers in the neonatal period had long-term effects on the biodiversity of their microbiomes that persisted into adulthood (164), and stressing adult rodents also causes an altered microbiome (181). Similarly, the microbiomes of severely stressed sick humans show prolonged changes (182). The mechanisms include changes in gut mobility and function and redirection of blood away from the gut mediated by signals from the vagus nerve and enteric nervous system (183), and stress causes release both systemically and in the gut of mediators such as catecholamines that modulate microbial growth (184). Moreover, since the immune system has a role in “farming” the microbiota, it is clear that stress-mediated changes in immune function will also impact the microbiota (166). There are many SES-associated stressors, such as drug abuse, violence, fear, heat, noise, poverty, and sleep disorders (Fig. 2). Some of these are outlined below.

### (i) Noise.

Noise is an important cause of stress, though its effects are not easily distinguished from other stressors that occur in low-SES neighborhoods such as pollution and poverty. Nevertheless, it is interesting that a major study of 504,271 individuals in Norway, the Netherlands, and the United Kingdom found that local road traffic noise was associated with markers of obesity (185).

### (ii) Heat.

An important review has summarized evidence that excessive heat caused 296,000 deaths globally in 2018, particularly in India and Indonesia but also in Europe (186). In low-SES contexts there are correlations between rises in temperature, increased hospitalizations, and risks of preterm births and stillbirths (187, 188). Individuals of low SES are less likely to benefit from air conditioning and may also suffer from the heat output from the air conditioners of their wealthier neighbors. Are these detrimental effects on health mediated via changes in the microbiota? It is difficult to disentangle the effects of heat from the effects of the psychological stress resulting from discomfort. Interestingly, heat stress reduced the biodiversity of the fecal microbiomes of cows and chickens (189, 190). Could similar effects occur in humans?

### (iii) Sleep disorders.

Sleep disturbance is associated with altered microbiota and decreased abundance of SCFA-producing strains both in humans (191) and in laboratory rodents (discussed in reference 192). The prevalence of sleep disturbances such as very short or long sleep correlated with SES in both African Americans and whites (193). Moreover, the direct involvement of the microbiota is suggested by the finding that fecal microbiota transplantation (FMT) from experimentally sleep-disturbed mice to normal mice can induce sleep disturbances in the recipients (192).

## CONCLUSIONS

Low SES is associated with illness and reduced life expectancy (1–3) and also with abnormal microbiomes (6–8). This paper summarizes evidence that these two observations are linked and that lifestyle factors that accompany low SES can reduce and distort microbial exposures and cause them to diverge from the exposures with which humans coevolved. This can explain at least part of the SES-associated health deficit. Awareness of the involvement of microbial exposures may enable better targeting of societal measures to improve the health of the deprived. These measures obviously include reduced pollution; better housing; better education about the value of breastfeeding, natural birth, and vaccines; encouragement of lifestyle adjustments that maximize exposure to nature; dietary guidance; and abandonment of broad-spectrum antibiotics in favor of antibiotics that target an identified pathogen. We now have the technology for this. Microbiological approaches will be particularly helpful for dietary supplementation with well-researched laboratory-grown communities of beneficial microorganisms as a safer, standardizable alternative to fecal microbiota transplantation (FMT). Unfortunately, designing such a standardized microbial preparation for FMT presents major challenges. Comparing the microbiomes of low- and high-SES individuals reveals differences but not a consistent high-SES-related composition, perhaps because the relevant studies span different methods, countries, diets, and cultural backgrounds. It is possible that the metabolic pathways (metabolome) provided by the gut microbiota are more relevant than the constituent species (194). Moreover, there may be more than one stable microbial composition that can yield an appropriate metabolome, and different microbial compositions might be stable in individuals with different genetic, immunological, and dietary backgrounds. We still have a lot to learn.

Microbiology will also help us to devise strategies for the greening of cities and homes. The selection of soils and plants for ubiquitous minigardens (https://theediblebusstop.com/) and the development of “bioreceptive” materials for building new homes (http://www.richard-beckett.com/) can be guided by increasing understanding of the organisms that we need to encounter. How, for example, do we ensure contact with the spores of human gut-adapted strains? It is worth noting that all the microbe-focused strategies suggested by this text overlap the 17 Sustainable Development Goals (SDG) of the United Nations (https://sdgs.un.org/goals), so perhaps microbiologists can provide additional impetus to the achievement of these goals.
